# [Retracted] uPAR and cathepsin B knockdown inhibits radiation-induced PKC integrated integrin signaling to the cytoskeleton of glioma-initiating cells

**DOI:** 10.3892/ijo.2026.5844

**Published:** 2026-01-05

**Authors:** Kiranmai Alapati, Sreelatha Gopinath, Rama Rao Malla, Venkata Ramesh Dasari, Jasti S. Rao

Int J Oncol 41: 599-610, 2012; DOI: 10.3892/ijo.2012.1496

Following the publication of the above paper, it was drawn to the Editor's attention by a concerned reader that, regarding the wound healing assay data shown in Fig. 2D, a pair of the data panels appeared to be overlapping, such that the data from the same original source had apparently been used to show the results of differently performed experiments. Furthermore, concerning the immunohistochemical data shown in Fig. 7A and C, similarly, two pairs of the data panels contained overlapping data, where the results of different experiments were intended to have been portrayed.

Owing to the duplications of data that were identified in this paper, the Editor of *International Journal of Oncology* has decided that it should be retracted from the Journal on account of a lack of confidence in the presented data. The authors were asked for an explanation to account for these concerns, but the Editorial Office did not receive a reply. The Editor apologizes to the readership for any inconvenience caused.

